# Use of DNA pools of a reference population for genomic selection of a binary trait in Atlantic salmon

**DOI:** 10.3389/fgene.2022.896774

**Published:** 2022-08-26

**Authors:** Binyam Dagnachew, Muhammad Luqman Aslam, Borghild Hillestad, Theo Meuwissen, Anna Sonesson

**Affiliations:** ^1^ Fisheries and Aquaculture Research, Nofima AS—Norwegian Institute of Food, Tromsø, Norway; ^2^ Benchmark Genetics, Bergen, Norway; ^3^ Norwegian University of Life Sciences, As, Norway

**Keywords:** genomic selection, DNA pooling, reference population, salmon, in-silico

## Abstract

Genomic selection has a great potential in aquaculture breeding since many important traits are not directly measured on the candidates themselves. However, its implementation has been hindered by staggering genotyping costs because of many individual genotypes. In this study, we explored the potential of DNA pooling for creating a reference population as a tool for genomic selection of a binary trait. Two datasets from the SalmoBreed population challenged with salmonid alphavirus, which causes pancreas disease, were used. Dataset-1, that includes 855 individuals (478 survivors and 377 dead), was used to develop four DNA pool samples (i.e., 2 pools each for dead and survival). Dataset-2 includes 914 individuals (435 survivors and 479 dead) belonging to 65 full-sibling families and was used to develop in-silico DNA pools. SNP effects from the pool data were calculated based on allele frequencies estimated from the pools and used to calculate genomic breeding values (GEBVs). The correlation between SNP effects estimated based on individual genotypes and pooled data increased from 0.3 to 0.912 when the number of pools increased from 1 to 200. A similar trend was also observed for the correlation between GEBVs, which increased from 0.84 to 0.976, as the number of pools per phenotype increased from 1 to 200. For dataset-1, the accuracy of prediction was 0.71 and 0.70 when the DNA pools were sequenced in 40× and 20×, respectively, compared to an accuracy of 0.73 for the SNP chip genotypes. For dataset-2, the accuracy of prediction increased from 0.574 to 0.691 when the number of in-silico DNA pools increased from 1 to 200. For this dataset, the accuracy of prediction using individual genotypes was 0.712. A limited effect of sequencing depth on the correlation of GEBVs and prediction accuracy was observed. Results showed that a large number of pools are required to achieve as good prediction as individual genotypes; however, alternative effective pooling strategies should be studied to reduce the number of pools without reducing the prediction power. Nevertheless, it is demonstrated that pooling of a reference population can be used as a tool to optimize between cost and accuracy of selection.

## 1 Introduction

Genomic selection (GS) is becoming a practical and effective breeding tool for many livestock species because of the rapid development of high-throughput genotyping technologies that reduce genotyping costs. The potential application of GS for aquaculture species has been studied (Nielsen et al., 2009; Sonesson and Meuwissen, 2009), and it showed an increase in genetic gain especially for traits that are difficult to improve by traditional selection such as disease resistance. Therefore, it is of particular interest in aquaculture species as most breeding goal traits in these species are measured on sibs of the selection candidates.

For a conventional GS breeding program, two large datasets are required: a training set (reference population) with genotyped and phenotyped individuals and a prediction set (selection candidates) containing only genotyped individuals (Meuwissen et al., 2001; Goddard and Hayes, 2007). The sizes of these datasets determine the rate of genetic improvement through influencing components of the equation. On one hand, the prediction accuracy relies on the size of the training set to estimate parameters (i.e., marker effects) and the marker density at which reference individuals are genotyped. On the other hand, the size of the prediction set determines the selection intensity and consequently the response to selection. However, increasing either the training set or the prediction set increases the cost of GS programs.

Even though the genotyping cost per individual is reducing, implementation of conventional GS is expensive in aquaculture compared to other livestock species because of the fact that the number of selection candidates and their siblings to genotype is large. Therefore, it is of interest to reduce either the number of individuals or the number of markers to genotype without reducing the prediction accuracy significantly. Strategies for reducing the number of markers have been described in many studies (Lillehammer et al., 2013; Ødegård and Meuwissen, 2014; Dagnachew and Meuwissen, 2019; Tsairidou et al., 2020). This study investigates the impact of reducing the number of individuals to genotype by pooling DNA samples from a reference population.

Pooling of DNA samples and sequencing have provided a cost-effective alternative for a wide range of genomic applications, such as population genetics (Gautier et al., 2013), genome-wide association studies (Sham et al., 2002), and estimation of SNP effects for quantitative traits (Henshall et al., 2012; Bell et al., 2017). In a theoretical way, estimation of marker effects from a pooled DNA differs from standard individual genotypes in some aspects. First, in pooled DNA samples, only marker allele frequencies can be estimated, whereas in standard genotyping, individual marker genotypes are obtained. Second, in pooled DNA samples, marker allele frequencies will normally be estimated with some degree of technical error, unequal contribution of sequenced reads derived among the individuals in a specific pool. Third, for DNA pools, the quantitative trait value of each individual cannot be assigned to a particular marker genotype, since information is not available on individual genotypes. However, by using the allelic frequencies at each tail to estimate the respective genotype frequencies and by assigning the sample average at each tail to every individual at that tail, the problems raised by the above differences can be overcome (Henshall et al., 2012; Gautier et al., 2013).

Sonesson et al. (2010) studied the use of DNA pooling of test individuals in combination with communal rearing of families as a means of reducing genotyping costs in aquaculture GS schemes using simulation. The study reported up to 0.88 accuracy of selection depending on the number of test individuals and the number of markers. However, to date, the potential of DNA pooling of a reference population for genomic prediction using data from practical breeding work is lacking. Therefore, in this study, we demonstrate the potential of DNA pooling for GS using both pooled DNA samples and *in-silico* DNA pooling. The effect of the number of pools and sequencing depth on the selection accuracy is studied. The study uses datasets generated for a breeding work to improve resistance against pancreas disease (PD).

## 2 Materials and methods

### 2.1 Datasets

Two datasets from the SalmoBreed breeding population of two year-classes (YC) were used for this study. Dataset-1 was from YC 2013, and dataset-2 was from YC 2015. The trait of interest was resistance to PD. PD is currently among the most economically important diseases in Norwegian Atlantic salmon production. It is caused by a salmonid alphavirus (SAV) of which SAV2 and SAV3 variants are found in Norway. These datasets were generated as part of the SalmoBreed’s annual challenge test for the practical breeding work to improve host resistance against PD. The first dataset was used for real DNA sample pooling and the other was used for *in-silico* pooling.

#### 2.1.1 Dataset-1: DNA pooling of samples

**FIGURE 1:**
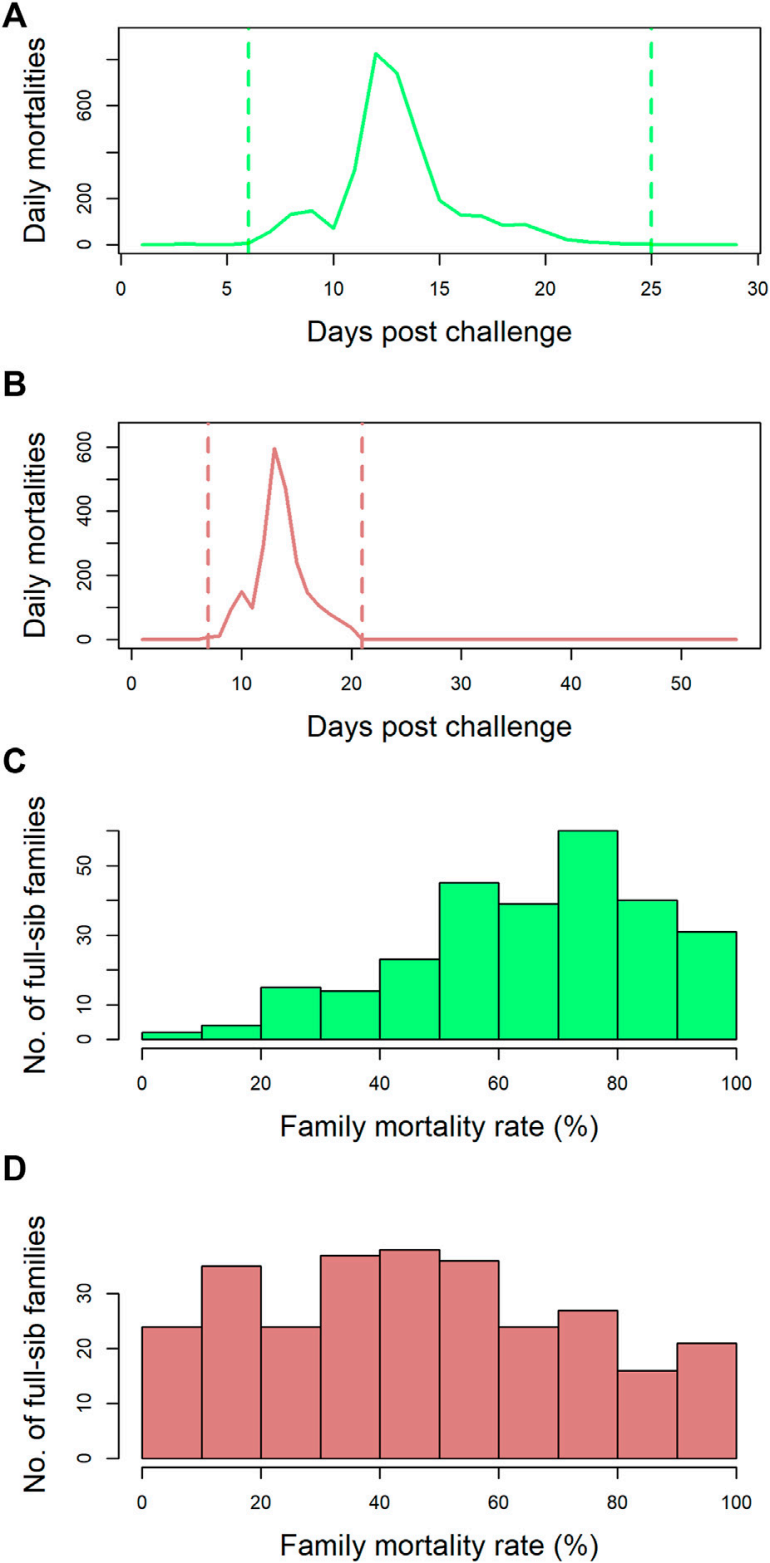
Mortality rate profiles of the datasets: **(A)** the number of mortalities observed per day over the course of the challenge trial (29 days) for dataset-1. Mortalities started 5 days post challenge and ended 25 days post challenge with the peak mortality observed at 12 days post challenge. **(B)** the number of mortalities observed per day over the course of the challenge trial (56 days) for dataset-2. Mortalities started 7 days post challenge and ended 21 days post challenge with the peak mortality observed at 13 days post challenge. **(C)** dataset-1—the number of full-sib families across the 273 full-sib families with a given percentage mortality. The mortality rate ranged from 0% to 100%, with an average mortality rate of 67%. **(D)** dataset-2—the number of full-sib families across the 282 full-sib families with a given percentage mortality. The mortality rate ranged from 0% to 100%, with an average mortality rate of 48%.

Data and tissue samples on 5,223 postsmolts belonging to 273 full-sib families from the SalmoBreed elite population challenged with SAV3 were available. The mortality profiles for this dataset are presented in Figures 1A and C. Mortalities started 5 days post challenge and ended 25 days post challenge with the peak mortality observed at 12 days post challenge (Figure 1A). A histogram of the number of full-sibling families across the 273 full-sib families with a given percentage of mortality is plotted in Figure 1C. The full-sibling mortality rate ranged from 0 to 100%, with an average mortality rate of 67% (Figure 1C). The individuals could be selected and grouped using survival information (early dead and/or late survivors) from the challenge test. Hence, 855 individuals were selected to develop four pools (i.e., M1, M2, S1, and S2 with the initial “M” representing the pool of mortalities/dead and “S” denoting the pool of surviving individuals).

DNA was extracted from each selected individual, quantified using the Quant-iT PicoGreen dsDNA assay kit, and normalized to a standard concentration for every individual; subsequently, equal quantities/volumes of DNA from each individual were pooled to make a specific pool group (M1, M2, S1, and S2). The M1 and M2 pools contained DNA representing 173 and 204 mortalities, respectively, while pools S1 and S2 incorporated DNA from 205 and 273 surviving individuals, respectively. The dead individuals of pools M1 and M2 represented 28 families, while pools S1 and S2 were represented by 35 and 34 families, respectively.

Libraries were prepared for sequencing using the Illumina PCR-free genomic DNA sample prep kits which were sequenced to approximately 40× depth. The sequencing was performed with an Illumina NextGen 500 instrument to obtain paired-end sequence reads of 150 bp. Trimmomatic software was used to perform adaptor and quality trimming of the generated sequence reads, and subsequently, high-quality sequence data were aligned to the Atlantic salmon genome reference sequence (assembly ICSASG_v2) using BWA-MEM version: 0.7.13-r1126 (Li, 2013). SNP detection, genotype calling, and allele frequencies on each locus were obtained using SAMtools version: 1.2. Software (Li et al., 2009). All the individuals pooled into four pools (M1, M2, S1, and S2) were also individually genotyped using ∼57 K axiom Affymetrix SNP Genotyping Array (NOFSAL2). The overlapping SNPs across genotyping methods (sequencing vs. axiom array) were identified which yielded 45812 SNPs in common that were used for further genomic analyses.

#### 2.1.2 Dataset-2: *In-silico* DNA pooling

Data from 4,115 postsmolts belonging to 282 full-sib families from the SalmoBreed elite population challenged with SAV3 were available. The mortality profiles for this dataset are presented in Figures 1B and D. Mortalities started 7 days post challenge and ended 21 days post challenge with peak mortality observed at 13 days post challenge (Figure 1B). A histogram of the number of full-sibling families across the 282 full-sib families with a given percentage of mortality is plotted in Figure 1D. The full-sibling mortality rate ranged from 0 to 100%, with an average mortality rate of 48% (Figure 1D). From the dataset, 914 individuals (435 survivors and 479 dead), belonging to 65 full-sib families, were selected based on family-wide mortality rates. The dead individuals represented 58 families, while the survived individuals represented 60 families. The data were split into a reference set (589 samples: 308 survivors and 281 dead) and a validation set (325 samples: 127 survivors and 198 dead). Splitting of individuals into reference and validation sets was performed randomly within a full-sib family; hence, each family was represented in both datasets.

Genotypes for the customized NOFSAL2 SNP array with ∼57 K SNPs were available for each individual. Pooled genotypes were generated in-silico as the frequency of alleles of the individuals in the pools and no random error was added to the pool genotypes.

### 2.2 Calculation of SNP allele frequencies

#### 2.2.1 Allele frequency in DNA pools (dataset-1)

The pools were sequenced on an average sequencing depth of ∼40× and the allele frequencies from each pool (S1, S2, M1, and M2) were obtained using a customized script implemented with *bcftools*, version: 1.10.2 (Danecek et al., 2021). The observed total allelic depth/frequency for each discovered variant in each pool was obtained using “*INFO/AD*” and the expression “*FORMAT/AD*” was used to obtain the observed frequency of the alternative allele. Moreover, the sequencing depth of each pool was reduced to 20× by random sampling of sequence reads followed by alignments, variants calling, and estimates of frequencies for both reference and nonreference alleles. The objective to reduce the sequencing depth per pool was to test the effect on the accuracy of prediction when the sequencing depth is reduced.

#### 2.2.2 Allele frequency in *in-silico* DNA pooling (dataset-2)

The reference set was used for the calculation of allele frequencies by in-silico pooling of the genotypes of individuals in this set. They were pooled into different numbers of pools (i.e., 1, 2, 4, 10, 20, 40, 100, 150, and 200) per phenotype group (i.e., dead and survivors). Summaries of the average number of individuals and families per pool and per phenotype group are presented in Table 1. Allele frequencies from each pool were calculated by sampling with replacement given the individual genotypes. The number of times the sampling with replacement was performed is related to the average sequence depth, and it was done 20×, 40×, and 100×. This process was repeated independently 60 times.

**TABLE 1:** Summary of the number of pools and the average number of individuals and families per pool per phenotype group.

# Pools	Dead pool		Alive pool	
	# Fish/pool	# Family/pool	# Fish/pool	# Family/pool
1	281	58	308	60
2	140.5	49.5	154	56
4	70.25	37.75	77	42.5
10	28.1	22.2	30.8	24.6
20	14.05	12.35	15.4	13.7
40	8.3	7.7	9.22	7.65
100	2.81	2.76	3.08	3.03
150	1.87	1.85	2.05	2.05
200	1.41	1.4	1.54	1.52

### 2.3 Estimation of marker effects

The main difference between individual genotypes and pooled DNA regarding estimation of marker effects is that for the latter, a trait value of individuals cannot be assigned to a particular marker genotype exclusively. Therefore, marker effects are to be estimated from marker allele frequencies calculated from pooled DNA.

For the individual genotypes, marker effects were estimated by fitting the marker-based genomic model (SNP-BLUP):

$$y_{ind}=1\mu +Xb_{ind}+e$$

where $y_{ind}$ is the vector of phenotypes for the trait, μ is the overall mean, 1 is the vector of 1s, *X* is the matrix of genotypes dosage for all SNP coded as 0,1, and 2 and for all animals, $b_{ind}$ is the vector of marker effects and it is assumed that $b_{ind}∼N\left(0,I\sigma_{m}^{2}\right)$ and  e  is the vector of random residual and assumed $e∼N\left(0,I\sigma_{e}^{2}\right)$ where $\sigma_{m}^{2}$ is the variance of marker effects, I is the identity matrix, and $\sigma_{e}^{2}$ is the residual error variance.

For the pools, the marker effects are estimated by fitting a slightly modified marker-based genomic model:

$$y_{pool}=1\mu +Zb_{pool}+e_{pool}$$

where $y_{pool}$ is the vector of phenotypes of the pools for the trait, μ is the overall mean, 1 is the vector of 1s, *Z* is the matrix of average allele frequencies for all SNPs and for all pools, $b_{pool}$ is the vector of marker effects estimated from allele frequencies of pools, and $e_{pool}$ is the vector of random residual. It is assumed that $b_{pool}∼N\left(0,I\sigma_{m-pool}^{2}\right)$ and $e_{pool}∼N\left(0,I\sigma_{e-pool}^{2}\right)$ , where $\sigma_{m-pool}^{2}$ is the variance of marker effects estimated from the marker frequencies of the DNA pools and $\sigma_{e-pool}^{2}$ is the residual error variance. The analyses for both models were done using singular value decomposition based SNP-BLUP (Ødegård et al., 2018), which is suitable for large-scale genomic predictions.

### 2.4 Estimation of genomic breeding values

Estimation of genomic breeding values (GEBVs) for the selection candidates was performed by summing the effects of the markers multiplied by the standardized genotypes. Two GEBV values per individual were predicted using SNP effects estimated using pool data and the individual genotypes.

$$GEBV_{j}=\overset{m}{\underset{i}{\sum }}X_{j}b_{i}$$

where $GEBV_{j}$ is the vector of predicted GEBVs for individual j, $X_{i}$ is the standardized genotypes for individual *j*, and $b_{i}$ is the calculated marker effects either from pools of samples or individual genotypes.

### 2.5 Model evaluation

For dataset-1 DNA pooling of samples, the effect of sequencing depth on the accuracy of selection was studied by varying the sampling from originally available 40× to 20×. SNP effects from the pool data were calculated based on allele frequencies estimated from the pools and used to calculate GEBVs as described in the estimation of GEBV section.

For dataset-2 in-silico DNA pooling, the effect of sequence coverage on the accuracy of selection was studied by varying the sampling times to 40× and 100×. SNP effects from the pool data were calculated based on allele frequencies estimated from the pools and compared with SNP effects estimated from individual genotypes.

The accuracy of selection was calculated as the correlation between predicted GEBVs and phenotypes and weighted by the square root of the heritability (*h* ^2^ = 0.3).

$$Accuracy\;\left(r_{corr}\right)=\frac{\rho \left(GEBV,y\right)}{\sqrt{h^{2}}}$$

Where ρ is the Pearson moment correlation coefficient, *GEBV* is the estimated GEBVs, y is the adjusted phenotype, and *h* ^2^ is the heritability of the trait.

## 3 Results

### 3.1 Accuracy of allele frequency estimation

**FIGURE 2:**
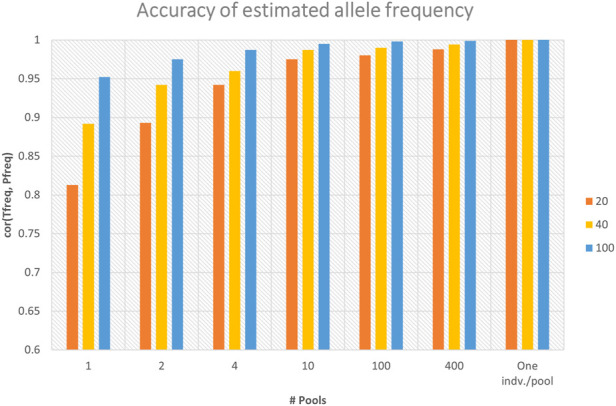
Accuracy of marker allele frequency calculated from different numbers of in-silico DNA pools and sequencing depths. Accuracy was calculated as the Pearson correlation coefficient between the true allele frequencies (“Tfreq”) (i.e., calculated from the individual genotypes) and frequencies calculated using in-silico DNA pools (“Pfreq”) (*n* = 1, 2, 4, 10, 100, and 914). There were 914 individuals, and the maximum number of pools (*n* = 914) represents one individual per pool.

Sequencing of DNA pools from individuals gives estimates of allele frequencies at SNPs. For dataset-1, the observed number of alleles at each locus for the sequenced pools provided the estimates of allele frequencies. The accuracies of marker allele frequency calculated from the in-silico DNA pools using three sequencing depths (i.e., 20×, 40×, and 100×) are presented in Figure 2. The accuracies were calculated as the Pearson correlation coefficients between the true allele frequencies (i.e., calculated from the individual genotypes) and frequencies calculated using in-silico pools. The figure shows that the accuracy of allele frequency estimation is affected by the number of pools and the average sequence depth coverage. As the number of pools increased from one pool, where all individuals represent one pool, to the maximum number of pools where each individual denotes a pool, the accuracy of allele frequency calculation has improved significantly, especially with low average sequence coverage (Figure 2). For example, for the sequencing coverage 40×, the Pearson correlation coefficients between DNA pool- and individual-based allele frequency estimation increased from 0.892 when only one DNA pool is considered to 0.99 when 10 DNA pools are used (Figure 2). Similar trends were also observed for the different sequencing coverages (Figure 2). As can be also seen from the figure, increasing sequence coverage depth also improved the accuracy of allele frequency estimation, particularly when the number of pools is small (Figure 2). When a single pool per individual was used (i.e., no. of pools = 914), the correlation between allele frequencies calculated using individual genotypes and the pool is equal to 1, regardless of the sequencing coverage.

**FIGURE 3:**
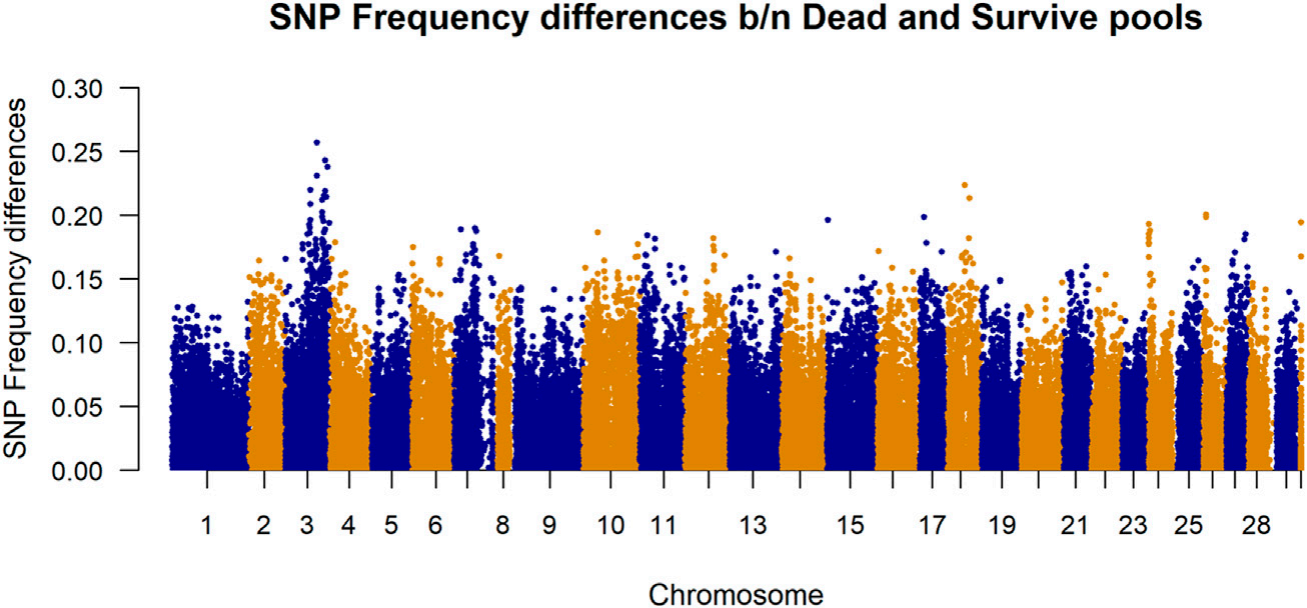
Manhattan plot of allele frequency differences between alive and dead pool for each SNP. The plotted allele frequency differences were calculated from 100 pools per phenotype group. Chromosome 30 represents markers belonging to unknown chromosome(s).

Differences in SNP frequencies between survivor and dead in-silico DNA pools are presented in Figure 3. The plotted allele frequency differences in the figure are absolute values of the frequency differences calculated from 100 pools per phenotype group. It shows that there are larger differences in frequencies between dead and survivor pools for the SNPs located at chromosome 3.

### 3.2 Correlation between SNP effects

**FIGURE 4:**
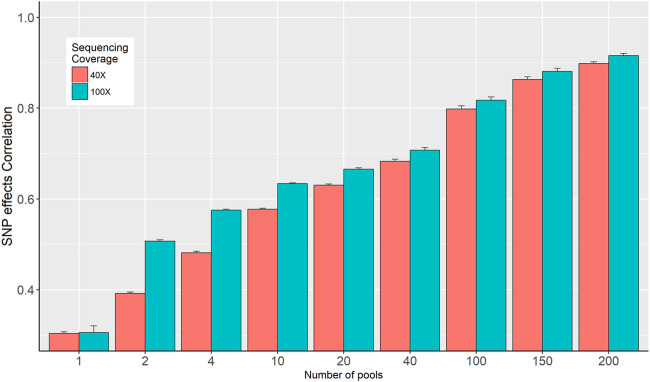
Correlation between SNP effects estimated from individual genotypes and in-silico DNA pool genotypes.

The correlations between SNP effects estimated from individual genotypes and based on allele frequencies calculated from the in-silico DNA pools are presented in Figure 4. The correlation increased from 0.3 to 0.898 and from 0.311 to 0.912 for the 40× and 100× sequence coverage respectively, when the number of pools increased from 1 to 200 per phenotype group (Figure 4). Overall, it was observed that the impact of sequencing coverage on the correlation between SNP effects is limited; however, its importance increases when the number of pools is decreasing. Exception from the general trend was observed when 1 pool per phenotype group was used, where no difference in SNP effects correlation was observed for 40× and 100× (Figure 4).

### 3.3 Correlation between genomic breeding values

**FIGURE 5:**
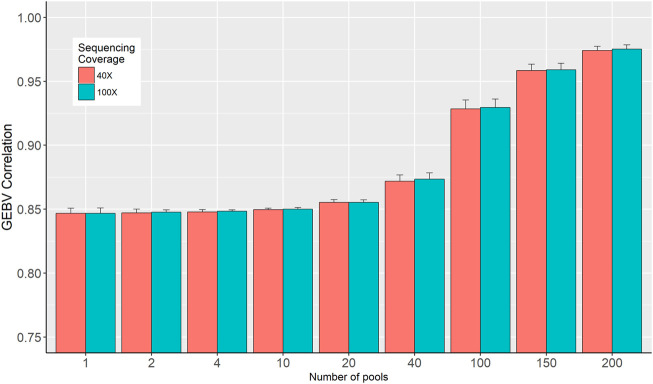
Correlation between genomic breeding values (GEBV) estimated from individual genotypes and in-silico DNA pool genotypes.

Two sets of GEBVs for the validation dataset were calculated based on SNP effects estimated from individual genotypes and in-silico pooled DNA. Pearson correlation coefficients between these two sets of GEBVs are presented in Figure 5. The figure shows that the number of pools is the determining factor as the correlation increased from 0.84, when only a single pool is used per phenotype, to 0.976 when the number of pools increased to 200 for 40× sequencing coverage (Figure 5). These correlations are barely affected by the sequence coverage.

### 3.4 Genomic prediction accuracy

For the validation dataset in dataset-2–in-silico DNA pooling, genomic prediction accuracies were calculated as the Pearson correlation coefficients between true phenotypes and predicted GEBVs and weighted by the inverse of the square root of heritability of PD (Table 2). The result showed that the prediction accuracy for the individual genotypes was 0.712 and for the in-silico DNA pools, it ranged from 0.574 to 0.687 when the number of pools increased from 1 to 100. Table 2 also presents % decreased, the decline in accuracy for the DNA pools compared to the individual genotypes. Regardless of the sequencing coverage, approximately a 20% decline in accuracy was observed for in-silico DNA pools when less than 10 pools per group were used. However, the loss in accuracy was reduced to less than 10% when 100 pools per phenotype group were used. Furthermore, less than a 4% loss in accuracy was observed when up to 200 pools were used. The difference between 40× and 100× sequencing coverage with respect to prediction accuracy was limited (Table 2).

**TABLE 2:** Accuracy of selection of dataset-2–in-silico DNA pooling.

No. of pools	Accuracy of prediction			% Decreased	
	Individual	40×	100×	40×	100×
1	0.712 ± 0.005	0.574 ± 0.006	0.575 ± 0.006	19.38	19.24
2		0.574 ± 0.004	0.575 ± 0.002	19.382	19.24
4		0.575 ± 0.003	0.575 ± 0.002	19.242	19.24
10		0.576 ± 0.003	0.576 ± 0.001	19.101	19.10
20		0.581 ± 0.005	0.581 ± 0.002	18.54	18.40
40		0.594 ± 0.008	0.596 ± 0.006	16.57	16.29
100		0.642 ± 0.009	0.641 ± 0.011	9.83	9.97
150		0.667 ± 0.012	0.671 ± 0.011	6.32	5.76
200		0.684 ± 0.012	0.687 ± 0.012	3.93	3.51

Accuracy of prediction using *in-silico* DNA pools of the reference population for different numbers of pools and sequencing coverage. The % decreased is the decrease in accuracy of prediction in % for the 40× and 100× compared to the individual genotype. The presented accuracies are the mean of 60 replicates and the standard errors are the standard deviation of 60 replicates.

The accuracy of prediction values for dataset-1–DNA pooling of samples and individual SNP chips are shown in Table 3. The accuracy of prediction was 0.737 for the individual SNP chip data, and 0.716 and 0.700 for pooled data when sequencing coverage was 40× and 20×, respectively. This is up to ∼5% higher accuracy for the individual SNP chip data.

**TABLE 3:** Accuracy of prediction for dataset-1–DNA pooling of samples.

Dataset	No. of SNPs	Accuracy of prediction
SNP chip	51646	0.737 ± 0.006
Sequence 40×	44538	0.716 ± 0.004
Sequence 20x	45812	0.700 ± 0.003

## 4 Discussion

Implementation of a conventional GS in aquaculture species is very expensive because of the very large number of selection candidates and test-sibs to be genotyped. In recent years, cost-efficient GS design approaches either to minimize the number of individuals or the number of markers to genotype, without significantly reducing the accuracy of selection, have been given emphasis (Lillehammer et al., 2013; Dagnachew and Meuwissen, 2019; Tsairidou et al., 2020). This study investigates the potential of DNA pooling for creating a reference population for genomic prediction of PD resistance (binary observation) in salmon. It demonstrated that SNP effects in a reference population can be estimated from SNP allele frequencies that are calculated from DNA pools and then the GEBVs for selection candidates can be economically computed with acceptable accuracies.

The datasets used in this study were generated as part of the SalmoBreed’s practical breeding work to improve host resistance against PD. PD is currently one of the most economically important diseases in the Norwegian production of Atlantic salmon (Jansen et al., 2010). The disease is caused by SAV, of which at least three distinct genotypes have been identified (Hodneland et al., 2005; Fringuelli et al., 2008). Mortality rates vary widely from PD outbreaks; survivors may eventually die because of secondary infections and increased parasitism and suffer reduced growth and degraded product quality (Lerfall et al., 2012). Moderate heritabilities have been reported from field outbreak data (Norris et al., 2008) and through controlled challenge testing using different challenge models (Gonen et al., 2015). Considerable efforts have been made in mapping genes for PD resistance for use in marker-assisted selection and a QTL for PD resistance was mapped to Atlantic salmon chromosome 3, using both fry and smolt challenge test data from two populations (Gonen et al., 2015; Hillestad et al., 2020). The difference in SNP frequencies between survivors and dead in-silico DNA pools (Figure 3) shows that there are larger differences in frequencies between dead and survivor pools for the SNPs located at chromosome 3. This validates that SNPs in that region are associated with some biological mechanisms which are likely to be influencing resistance to PD.

A sequence of DNA pools from individuals gives estimates of allele frequencies at SNPs with small or no loss in accuracy for a considerably lower cost compared to individual genotyping. Estimation of marker effects relies heavily on the accuracy of allele frequencies calculated from DNA pools. The accuracy of allele frequency estimation from pooled DNA samples depends on some experimental design parameters (Gautier et al., 2013), such as the number of individuals merged in a pool, the sequencing coverage, and the possibility of unequal contribution of each individual genome to the final sequencing read. The effects of the number of individuals merged in a pool were studied by varying the number of individuals in the pools (Table 1). However, the effect of sequencing coverage was studied by changing the number of sampling times (i.e., average sequencing coverage) for the in-silico pools and varying the sequencing coverage for pooled DNA samples. However, the effect of unequal contribution of individuals is not assessed in this study. It is important to note that accurate equimolar pooling of each genomic DNA is important for equal distribution of reads (Konczal et al., 2014) and the number of pooled samples should be balanced for accurate allele frequency estimation (Gautier et al., 2013; Konczal et al., 2014) and consequently, the implication of unequal DNA contribution for genomic prediction accuracy should be investigated.

For allele frequency calculation and SNP effects estimation, the importance of sequencing coverage decreased as the number of DNA pools increased (Figures 2 and 4). Given a fixed number of samples, as the number of pools increased, the number of individuals per pool reduced (Table 1), and thus the effectiveness of high sequencing coverage has diminished. This observed pattern is in agreement with that of Rellstab et al. (Rellstab et al., 2013), who reported that higher sequencing coverages (>50) have no significant effect on allele estimation accuracy and only very low coverages (below 20×) would substantially reduce the precision. In the current study, an exception from the general trend was when 1 pool per phenotype group was used, where the advantage of sequencing coverage was visible for allele frequency accuracy (Figure 2) but not for the correlation of SNP effects (Figure 4). Furthermore, the SNP effect correlations were poor for both 40× and 100× coverage.

The accuracy of GS is expected to increase as the number of genotyped and phenotyped animals in the reference population increases for any trait, in particular, for lowly heritable traits (Solberg et al., 2008). Our results showed that SNP effects correlation, GEBV correlations, and prediction accuracy increased as the number of pools increased from 1 to 200 per phenotype group (Figures 4 and 5; Table 2). Henshall et al. (012) showed that a large number of smaller pools would estimate allele frequency more accurately than small numbers of large pools. As the number of pools increased, the accuracy of allele frequency estimation increased (Figure 2) and the prediction accuracy also increased (Table 2). Moreover, for a larger number of pools, it is observed that there are very little or no differences in SNP frequency accuracies and SNP effects correlation among different sequencing coverages (Figures 2 and 4). Similar trends were reported (Henshall et al., 2012; Bell et al., 2017) when there are a large number of pools. Furthermore, the high correlations between GEBVs estimated from individual genotype and DNA pools, particularly for the large number of pools (Figure 5), evidenced that there is limited to no reranking of individuals.

The accuracy of breeding values using pedigree information for dataset-2 (only using phenotypes of 914 individuals and their pedigree) was 0.48 (the result is not presented). This accuracy was significantly improved by the use of genomic information from the pools, which is in agreement with other reports (Sonesson et al., 2010; Dagnachew and Meuwissen, 2019; Kriaridou et al., 2020; Tsairidou et al., 2020), especially for the large number of pools. The loss of accuracy for the use of DNA pools compared with the individual genotypes was minimal for dataset-1 (0.74 vs. 0.71, Table 3). However, the prediction accuracy difference was substantial for dataset-2 (0.71 vs. 0.57, Table 2) for the same number of pools. One explanation is that for dataset-1, the prediction accuracies were obtained for the same individuals in the pools (i.e., reference and validation were the same individuals). On the other hand, for dataset-2, the validation individuals were different from the reference population. Furthermore, computer simulation of DNA pooling provides an approximation and might fail to capture some parameters.

Results from using DNA pooling for genomic prediction are lacking. Our results show a trade-off between the number of DNA pools and the loss of prediction accuracy. A reduction in prediction accuracy means a reduction in genetic gain. This has a cost implication that is complex to quantify as it is determined by the trait, the breeding goal, and other specifics of the breeding industry. Assuming $20 genotyping cost per individual and $300 cost of sequencing per sample for a 40× sequencing depth, the annual genotyping cost for 5,000 individuals (1,000 candidates and 4,000 informant sibs) is $100,000. However, for the DNA pool scenario, where only 1,000 candidates are genotyped and the reference siblings are pooled, the cost of genotyping varies from $20,600 to $140,000 for a single pool per phenotype to 200 pools per phenotype, respectively. Increasing the number of pools also increases the cost of sequencing and hence an appropriate pooling strategy should strike an optimal balance between cost-effectiveness and accuracy. Furthermore, in the present study, we have presented the prediction of allele frequencies from the pools of DNA using sequencing of the pools. However, calculation of allele frequencies from pooled DNA does not necessarily require sequencing of the pools. It has been reported that allele frequencies from DNA pools can also be calculated by SNP genotyping of the pools using light intensities (Reverter et al., 2014); hence, the cost associated with sequencing of the pools can be avoided.

As it is presented in the study, DNA pooling of a reference population can serve as a cost-effective GS approach, but with a potential limitation in that the identity of individuals would be lost and therefore individual characteristics and environmental factors could not be adjusted in genomic modeling, which may result in a loss in accuracy and a biased estimate of a genetic effect. In the current study, pooling within phenotype groups was done randomly; however, Henshall et al. (2012) suggested that pooling strategies within contemporary groups and fitting contemporary group in the model would eliminate some of these limitations. For example, in the studied datasets, pooling within sex and full-sib families would address these limitations.

## 5 Conclusion

DNA pooling of a reference population can serve as a cost-effective GS approach, but with some potential limitations. Results showed that a large number of pools are required to achieve as good genomic prediction accuracies as individual genotypes; however, alternative effective pooling strategies should be exploited to reduce the number of pools without reducing the prediction power. Nevertheless, it is demonstrated that pooling of a reference population can be used as a tool to optimize between cost and accuracy of selection.

## Data Availability

The datasets presented in this article are not readily available because it is co-owned by a third party. Request to access the datasets should be directed to anna.sonesson@nofima.no and hooman.moghadam@bmkgenetics.com.
